# Supplementary material for: Plasma Phospholipid Fatty Acids, FADS1 and Risk of 15 Cardiovascular Diseases: A Mendelian Randomisation Study
Source: Nutrients. 2019 Dec 7;11(12):3001. doi: 10.3390/nu11123001 (PMC6950527; doi:10.3390/nu11123001)
Supplement: Supplementary file 1 [file nutrients-11-03001-s001.pdf]

Supplementary Table 1. Information of included studies and consortia

Exposure/Outcome	Consortium or cohort study	Participants	Web source if publicly available
n-3 PUFAs	ARIC, CHS, CARDIA, InCHIANTI and MESA studies	8866 individuals of European ancestry	Not available
n-6 PUFAs	ARIC, CHS, CARDIA, InCHIANTI and MESA studies	8631 individuals of European ancestry	Not available
n-7 MUFA, n-9 MUFA and SFA	ARIC, CHS, CARDIA, InCHIANTI and MESA studies	8961 individuals of European ancestry	Not available
Coronary artery disease	CARDIoGRAMplusC4D consortium	184 305 individuals (60 801 coronary artery disease cases and 123 504 non-cases) of mainly European (77%) and Asian (19%) ancestry	www.cardiogramplusc4d.org/
Atrial fibrillation	Atrial Fibrillation Consortium	588 190 individuals (65 446 atrial fibrillation cases and 522 744 non-cases) of mainly European (91%) and Asian (6%) ancestry	http://www.broadcvdi.org
Ischemic stroke and subtypes	MEGASTROKE consortium	514 791 individuals (60 341 ischemic stroke cases and 454 450 non-cases) of mainly European (86%) and Asian (9%) ancestry; 6688 large artery stroke cases, 11 710 small vessel stroke cases and 9006 cardioembolic stroke cases	http://www.megastroke.org/
Nine cardiovascular diseases (see Supplementary Table 3)	UK Biobank	367 643 unrelated European-descent individuals	https://www.ukbiobank.ac.uk/

ARIC indicates Atherosclerosis in Risk in Communities; CARDIA, Coronary Artery Risk Development in Young Adults; CHS, Cardiovascular Health Study; InCHIANTI, Invecchiare in Chianti; MESA, Multi-Ethnic Study of Atherosclerosis; MUFA, monounsaturated fatty acid; PUFA, polyunsaturated fatty acids; SFA, saturated fatty acid.

Supplementary Table 2. Definitions and sources of information for CAD, AF and ischemic stroke and its subtypes from consortia

Outcome	Consortium	Cases	Non-cases	Case definition	Source
CAD	CARDIoGRAMplusC4D	60 801	123 504	Case status was defined by an inclusive CAD diagnosis (e.g. myocardial infarction, acute coronary syndrome, chronic stable angina, or coronary stenosis >50%)	http://www.cardiogramplusc4d.org
AF	Atrial Fibrillation Consortium	65 446	522 744	AF was defined as paroxysmal or permanent AF or atrial flutter	www.broadcvdi.org
AIS	MEGASTROKE	60 341	454 450	The classification of subtypes was mainly based on the Trial of Org 10172 in Acute Stroke Treatment (TOAST) criteria	http://www.megastroke.org/
LAS	MEGASTROKE	6688	454 450		
CES	MEGASTROKE	11 710	454 450		
SVS	MEGASTROKE	9006	454 450		

AF indicates atrial fibrillation; AIS, any ischemic stroke; CAD, coronary artery disease; CES, cardioembolic stroke; LAS, large artery stroke; SVS, small vessel stroke.

Supplementary Table 3. Definitions and sources of information for cardiovascular disease outcomes in UK Biobank

Outcome	Number of cases	ICD-9 diagnosis	ICD-10 diagnosis	OPCS procedure	Self-report†
Heart failure	6712	402.01, 402.11, 402.91, 404.01, 404.11, 404.91, 404.03, 404.13, 404.93, 428	I11.0, I13.0, I13.2, I50		20002
Aortic valve stenosis	2244		I35.0, I35.2		20002
Abdominal aneurysm	1094	441.3, 441.4	I71.3, I71.4	L19.4, L19.5	20002
Thoracic aneurysm	347	441.1, 441.2	I71.1, I71.2		20002
Transient ischemic attack	3962	435	G45		20002
Intracerebral hemorrhage	1064	431	I61		20002
Subarachnoid hemorrhage	1084	430	I60		20002
Venous thromboembolism	15602	415.1, 451.1, 452, 453.0, 453.4, 453.9	I26, I80.1, I80.2, I81, I82.0	L90.2	20002, 6152
Peripheral arterial disease	3415	443.8, 443.9	I73.8, I73.9		20002

ICD, International Classification of Disease; OPCS, Office of Population Censuses and Surveys Classification of Surgical Operations and Procedures.
Follow-up for incident cases was until March 31, 2017 and date of death was recorded until February 14, 2018.
†Numbers refer to data codes used in UK Biobank: 6150/6152 = Health condition diagnosed by doctor (self-reported); 6177 = Medication for health condition (self-reported); 20002 = Non-cancer illness code (self-reported from interview with nurse); 20004 = Surgical operation code (self-reported from interview with nurse).

Supplementary Table 4. Single-nucleotide polymorphisms selected as instrumental variables for individual FAs

Type of FA	FA	SD [a]	SNP	Chr	Nearby gene	EA	% variance explained	Summary statistics for SNP-FA association		
								Beta [b]	SE	P
n-3 PUFA	ALA	0.05	rs174547	11	*FADS1*	C	1	0.02	0.001	3.50E-64
	EPA	0.3	rs3798713	6	*ELOVL2*	C	0.4	0.04	0.005	1.90E-12
			rs174538	11	*FADS1/C11orf10*	G	1.7	0.08	0.005	5.40E-58
	DPA	0.17	rs780094*	2	*GCKR*	T	0.5	0.02	0.003	9.00E-09
			rs3734398	6	*ELOVL2*	C	2.7	0.04	0.003	9.70E-43
			rs174547	11	*FADS1*	T	8.4	0.08	0.003	3.80E-154
	DHA	0.89	rs2236212	6	*ELOVL2*	G	0.7	0.11	0.014	1.30E-15
n-6 PUFA	LA	2.69	rs10740118	10	*JMJD1C*	G	0.2-0.7	0.25	0.050	8.10E-09
			rs174547	11	*FADS1*	C	7.6-18.1	1.47	0.050	5.00E-274
			rs16966952	16	*NTAN1*	G	0.5-2.5	0.35	0.040	1.20E-15
	AA	1.96	rs174547	11	*FADS1*	T	3.7-37.6	1.69	0.020	3.3E-971
			rs16966952	16	*NTAN1*	G	0.1-0.6	0.20	0.030	2.40E-10
n-7 MUFA	POA	0.18	rs780093*	2	*GCKR*	T	0.2-0.9	0.02	0.003	9.80E-10
			rs6722456	2	*RN7SKP93*	G	0.01-0.6	0.05	0.009	4.10E-08
			rs603424	10	*SCD/PKD2L1*	G	0.3-1.6	0.03	0.004	5.70E-15
			rs11190604	10	*HIF1AN*	G	0.02-0.7	0.02	0.004	5.70E-09
			rs102275	11	*FADS1/2*	C	0.15-1.0	0.02	0.003	6.60E-13
n-9 MUFA	OA	1.17	rs102275	11	*FADS1/2*	C	0.3-2.1	0.23	0.020	2.20E-32
SFA	PA	1.64	rs2391388	1	*ALG14*	C	0.2-1.0	0.18	0.030	2.70E-11
	SA	1.19	rs6675668	1	*ALG14*	G	0.4-1.4	0.17	0.020	2.20E-18
			rs11119805	1	*LPGAT1*	T	0.01-0.7	0.17	0.030	2.80E-09
			rs102275	11	*FADS1/2*	T	0.3-1.2	0.18	0.020	1.30E-20

AA indicates arachidonic acid; ALA, α-linolenic acid; Chr, chromosome; DHA, docosahexaenoic acid; DPA, docosapentaenoic acid; EA, effect allele; EAF, effect allele frequency; EPA, eicosapentaenoic acid; FA, fatty acid; LA, linoleic acid; LAS, larger artery stroke; MUFA, monounsaturated fatty acid; OA, oleic

acid; PA, palmitic acid; POA, palmitoleic acid; PUFA, polyunsaturated fatty acid; SA, stearic acid; SD, standard deviation; SE, standard error; SFA, saturated fatty acid; SNP, single-nucleotide polymorphisms.

[a] The unit for SD is the percentage of total FAs.

[b] The beta coefficients represent the change in percentage of total fatty acids for each additional effect allele.

*SNPs in *GCKR* gene were excluded from all analyses due to multiple pleiotropic associations with potential confounders.

Supplementary Table 5. Related traits of the single-nucleotide polymorphisms associated with plasma fatty acid levels from PhenoScanner search

FA	SNP	EA	Nearby gene	Related trait	Beta	P	Related trait	Beta	P	Related trait	Beta	P
ALA	rs174547	C	*FADS1*	Triglycerides	0.05	1.04E-40	Red cell distribution width	-0.05	6.77E-40	Low density lipoprotein	-0.05	7.99E-38
EPA	rs174538	G	*FADS1/C11orf10*	Red cell distribution width	0.05	1.55E-43	Low density lipoprotein	0.05	1.07E-34	Pulse rate	-0.03	2.34E-32
DPA	rs780094	T	*GCKR*	Triglycerides	0.11	2.65E-220	Total cholesterol	0.05	5.28E-41	Alcohol intake frequency	0.05	1.13E-39
DPA	rs174547	T	*FADS1*	Triglycerides	0.05	1.04E-40	Red cell distribution width	-0.05	6.77E-40	Low density lipoprotein	-0.05	7.99E-38
LA	rs10740118	G	*JMJD1C*	Mean platelet volume	0.13	7.26E-286	Platelet count	-0.08	2.51E-95	Triglycerides	0.03	1.81E-11
LA	rs174547	C	*FADS1*	Triglycerides	0.05	1.04E-40	Red cell distribution width	-0.05	6.77E-40	Low density lipoprotein	-0.05	7.99E-38
LA	rs16966952	G	*NTAN1*	Whole body fat-free mass	0.01	3.67E-11	Basal metabolic rate	0.01	9.52E-11	Height	0.01	2.00E-10
AA	rs174547	T	*FADS1*	Triglycerides	0.05	1.04E-40	Red cell distribution width	-0.05	6.77E-40	Low density lipoprotein	-0.05	7.99E-38
AA	rs16966952	G	*NTAN1*	Whole body fat-free mass	0.01	3.67E-11	Basal metabolic rate	0.01	9.52E-11	Height	0.01	2.00E-10
POA	rs780093	T	*GCKR*	Triglycerides	0.11	2.65E-220	Total cholesterol	0.05	5.28E-41	Alcohol intake frequency	0.05	1.13E-39
POA	rs603424	G	*SCD/PKD2L1*	Blood metabolite ratios	-0.05	1.00E-115	Mean platelet volume	0.06	4.56E-31	Heel bone mineral density	0.03	3.77E-12
POA	rs11190604	G	*HIF1AN*	Leg predicted mass left	-0.01	4.16E-11	Whole body fat-free mass	-0.01	7.48E-10	Height	-0.01	9.60E-10
POA	rs102275	C	*FADS1/2*	Red cell distribution width	-0.05	4.49E-41	Triglycerides	0.05	4.27E-40	Low density lipoprotein	-0.05	7.61E-40
OA	rs102275	C	*FADS1/2*	Red cell distribution width	-0.05	4.49E-41	Triglycerides	0.05	4.27E-40	Low density lipoprotein	-0.05	7.61E-40
SA	rs102275	T	*FADS1/2*	Red cell distribution width	-0.05	4.49E-41	Triglycerides	0.05	4.27E-40	Low density lipoprotein	-0.05	7.61E-40

Supplementary Table 5 (cont.). Related traits of the single-nucleotide polymorphisms associated with plasma fatty acid levels from PhenoScanner search

FA	SNP	EA	Nearby gene	Related trait	Beta	P	Related trait	Beta	P	Related trait	Beta	P
ALA	rs174547	C	*FADS1*	Pulse rate	0.03	8.59E-38	Total cholesterol	-0.05	1.35E-35	Mean platelet volume	-0.04	1.14E-24
EPA	rs174538	G	*FADS1/C11orf10*	Mean platelet volume	0.04	1.72E-24	Triglycerides	-0.04	1.47E-19	Height	0.01	2.63E-12
DPA	rs780094	T	*GCKR*	Fat-free mass	-0.02	4.92E-39	Serum urate	0.08	6.52E-39	Monocyte percentage of white cells	-0.04	4.02E-34
DPA	rs174547	T	*FADS1*	Pulse rate	0.03	8.59E-38	Total cholesterol	-0.05	1.35E-35	Mean platelet volume	-0.04	1.14E-24
LA	rs10740118	G	*JMJD1C*	Average weekly beer plus cider intake	0.01	2.28E-09	Body mass index	0.01	1.05E-08	Years of educational attainment	-0.01	1.36E-08
LA	rs174547	C	*FADS1*	Pulse rate	0.03	8.59E-38	Total cholesterol	-0.05	1.35E-35	Mean platelet volume	-0.04	1.14E-24
LA	rs16966952	G	*NTAN1*	Weight	0.01	3.50E-08	Leg fat-free mass left	0.01	2.35E-10	NA	NA	NA
AA	rs174547	T	*FADS1*	Pulse rate	0.03	8.59E-38	Total cholesterol	-0.05	1.35E-35	Mean platelet volume	-0.04	1.14E-24
AA	rs16966952	G	*NTAN1*	Weight	0.01	3.50E-08	Leg fat-free mass left	0.01	2.35E-10	NA	NA	NA
POA	rs780093	T	*GCKR*	Fat-free mass	-0.02	4.92E-39	Serum urate	0.08	6.52E-39	Monocyte percentage of white cells	-0.04	4.02E-34
POA	rs603424	G	*SCD/PKD2L1*	Impedance of arm left	0.02	4.35E-12	Self-reported high cholesterol	-0.01	5.28E-08	NA	NA	NA
POA	rs11190604	G	*HIF1AN*	Basal metabolic rate	-0.01	1.09E-09	NA	NA	NA	NA	NA	NA
POA	rs102275	C	*FADS1/2*	Pulse rate	0.03	9.80E-37	High density lipoprotein	-0.04	6.40E-28	Triglycerides	0.05	1.58E-23
OA	rs102275	C	*FADS1/2*	Pulse rate	0.03	9.80E-37	High density lipoprotein	-0.04	6.40E-28	Triglycerides	0.05	1.58E-23
SA	rs102275	T	*FADS1/2*	Pulse rate	0.03	9.80E-37	High density lipoprotein	-0.04	6.40E-28	Triglycerides	0.05	1.58E-23

Supplementary Table 5 (continue). Related traits of the single-nucleotide polymorphisms associated with plasma fatty acid levels from PhenoScanner search

FA	SNP	EA	Nearby gene	Beta	P	Related trait	Beta	P
ALA	rs174547	C	*FADS1*	-0.02	2.10E-08	Wine intake	0.01	2.62E-08
EPA	rs174538	G	*FADS1/C11orf10*	-0.01	3.49E-08	NA	NA	NA
DPA	rs780094	T	*GCKR*	0.03	6.81E-27	Height	-0.02	1.91E-19
DPA	rs174547	T	*FADS1*	-0.02	2.10E-08	Wine intake	0.01	2.62E-08
LA	rs10740118	G	*JMJD1C*	-0.01	6.49E-08	Sodium in urine	0.01	2.52E-07
LA	rs174547	C	*FADS1*	-0.02	2.10E-08	Wine intake	0.01	2.62E-08
AA	rs174547	T	*FADS1*	-0.02	2.10E-08	Wine intake	0.01	2.62E-08
POA	rs780093	T	*GCKR*	0.03	6.81E-27	Height	-0.02	1.91E-19
POA	rs102275	C	*FADS1/2*	-0.02	4.97E-17	Height	-0.01	1.89E-11
OA	rs102275	C	*FADS1/2*	-0.02	4.97E-17	Height	-0.01	1.89E-11
SA	rs102275	T	*FADS1/2*	-0.02	4.97E-17	Height	-0.01	1.89E-11

EA indicates effect allele; FAs, fatty acids; NA, not available; SNP, single-nucleotide polymorphisms.
Web of PhenoScanner V2: http://www.phenoscanner.medschl.cam.ac.uk/
